# Deficient Heart Function Syndrome: a new nursing diagnosis for people with heart failure

**DOI:** 10.1590/0034-7167-2024-0653

**Published:** 2025-10-03

**Authors:** Ana Paula Dias de Oliveira, Marcos Venícios de Oliveira Lopes, Rita de Cassia Gengo e Silva Butcher, Viviane Martins da Silva, Vinicius Batista Santos, Juliana de Lima Lopes, Alba Lucia Bottura Leite de Barros

**Affiliations:** I Universidade Federal de São Paulo. São Paulo, São Paulo, Brazil; II Universidade Federal do Ceará. Fortaleza, Ceará, Brazil; III Connell School of Nursing. Chestnut, United States of America

**Keywords:** Syndrome, Heart Failure, Nursing Diagnosis, Nursing Process, Standardized Nursing Terminology., Síndrome, Insuficiencia Cardíaca, Diagnóstico de Enfermería, Proceso de Enfermería, Terminología Normalizada de Enfermería.

## Abstract

**Objectives:**

to identify whether the set of nursing diagnoses “Excessive fluid volume”, “Decreased cardiac output”, “Decreased activity tolerance”, “Fatigue” and “Impaired comfort” constitutes the “Deficient Cardiac Function Syndrome” in people with heart failure.

**Methods:**

a cross-sectional analytical study, which included 360 participants who were assessed by a cardiology nurse who used the nursing process and NANDA-International classification. Latent class analysis and log-linear models were used to determine the occurrence of nursing diagnoses by cluster.

**Results:**

“Fatigue” and “Excessive fluid volume” had the highest sensitivity; “Decreased cardiac output” and “Impaired comfort” had the highest specificity; “Decreased activity tolerance” had similar sensitivity and specificity. Latent class analysis for the new diagnosis included all diagnoses. Nine combinations of diagnostic statuses exhibited conditional dependence.

**Conclusions:**

the concomitant presence and strong relationship between diagnoses provide evidence for the existence of a syndromic diagnosis in this population.

## RESUMEN

**Objetivos:**

identificar si el conjunto de diagnósticos de enfermería “Volumen excesivo de líquidos”, “Disminución del gasto cardíaco”, “Disminución de la tolerancia a la actividad”, “Fatiga” y “Alteración del confort” constituyen un “Síndrome de Función Cardíaca Deficiente” en personas con insuficiencia cardíaca.

**Métodos:**

estudio analítico transversal, que incluyó a 360 participantes quienes fueron evaluados por una enfermera especialista en cardiología que utilizó el proceso y clasificación de enfermería NANDA-Internacional. Se utilizaron análisis de clases latentes y modelos log-lineales para determinar la ocurrencia de diagnósticos de enfermería por cluster.

**Resultados:**

“Fatiga” y “Volumen excesivo de líquido” mostraron mayor sensibilidad; “Disminución del gasto cardíaco” y “Confort deteriorado” mostraron mayor especificidad; “tolerancia a la actividad disminuida” mostró una sensibilidad y especificidad similares. El análisis de clases latentes para el nuevo diagnóstico incluyó todos los diagnósticos. Nueve combinaciones de estados diagnósticos exhibieron dependencia condicional.

**Conclusiones:**

la presencia concomitante y la fuerte relación entre los diagnósticos evidencian la existencia del diagnóstico sindrómico en esta población.

## INTRODUCTION

Heart failure (HF) is a highly prevalent and debilitating health condition^(1)^ that involves complex pathological processes that affect multiple dimensions of a person’s life^(2)^. As a result, individuals with HF experience multiple human responses simultaneously^(3)^, represented by clusters of nursing diagnoses (NDs). These clusters are characterized as a syndrome if they comprise at least two NANDA-International (NANDA-I)-approved diagnoses and the same interventions are used to treat them^(4)^.

According to a literature review, people with HF often experience several NDs simultaneously, which are often diagnosed together by nurses in clinical practice, and for this reason, they were selected to compose the syndromic diagnosis of this study. These NDs include “Fatigue” (FA), “Excessive fluid volume” (EFV), “Decreased activity tolerance” (DAT) and “Decreased cardiac output” (DCO)^(5)^. The expanded understanding of the term “syndrome” as a multidimensional condition influenced the inclusion of the “Impaired comfort” (IC) ND, with the purpose of meeting the desire to compose a ND that identified compromised emotional and social elements, in addition to physical elements.

EFV is included in domain 2 (nutrition) and class 5 (hydration); DCO, DAT and FA are included in domain 4 (activity/rest), the first two diagnoses being in class 4 (cardiovascular/pulmonary responses), and the third in class 3 (energy balance). Finally, IC is included in domain 12 (comfort) and class 1 (physical comfort).

The occurrence of these NDs as a cluster is believed to be the result of a common pathophysiological process (poor cardiac function) that can be addressed with similar interventions. Thus, altered cardiac muscle function result in the activation of adaptive responses that trigger poor tissue perfusion^(1)^ (correlated with DCO) and volume overload^(1)^ (correlated with EFV). Furthermore, hypoperfusion culminates in the sustained perception of insufficient energy to perform daily activities^(1)^ (correlated with DAT) and a disabling sensation of physical and mental exhaustion^(1)^ (correlated with FA). In addition to physical discomfort, negative feelings arise from frequent hospitalizations, dependence on caregivers, and interruption of work activities^(6)^ (correlated with HF). Still, none of the NDs of syndrome approved in the classification (2018-2020)^(4)^, current edition, at the time of the beginning of the study, seemed adequate to accurately document the human responses presented by people with decompensated HF.

Therefore, the “Deficient Cardiac Function Syndrome” ND was developed based on scientific literature in health and the clinical experience of the authors of this study. It was defined as “physical, emotional, psychosocial, and spiritual distress due to a decrease in the heart’s ability to pump blood to the body and to the adaptive responses to the insulted heart muscle”. The defining characteristics (DCs) of the syndromic ND are FA, EFV, DAT, IC, and DCO. The related factor comprises structural and/or functional cardiac dysfunction.

## OBJECTIVES

To identify whether the set of NDs FA, EFV, DAT, IC and DCO constitutes the “Deficient Cardiac Function Syndrome” in people with HF.

## METHODS

### Ethical aspects

This study was conducted in accordance with ethical principles and was approved by the Research Ethics Committee. The Informed Consent Form (ICF) was obtained from all individuals involved in writing, and data confidentiality was ensured^(7)^.

### Study design, period and place

This is an analytical cross-sectional study that followed the STrengthening the Reporting of OBservational studies in Epidemiology guidelines^(8)^. Data collection was carried out between April 2019 and May 2022, in a public emergency room (ER) specialized in cardiovascular conditions, in São Paulo, Brazil.

### Population or sample; inclusion and exclusion criteria

Sample size was established considering the latent class analysis (LCA) technique for inference of each ND individually^(9)^. DCO was selected to define sample size, as it has the largest number of DC (n=36)^(4)^. A proportion of ten participants per DC determined a sample size at 360.

Participants with a medical diagnosis of decompensated HF, aged 18 years or older, admitted to the ER for no more than six hours, and with New York Heart Association functional classes (FCs) III and IV^(10)^ were included. Participants with reduced level of consciousness and/or confusion, hemodynamic instability, and those who required tracheal intubation during data collection were excluded.

### Study protocol

All individuals admitted to the ER with decompensated HF who met the eligibility criteria were invited to participate in the study and signed the ICF.

Sociodemographic (age, sex, race, marital status, religion, education, and occupation) and clinical data (previous and current health history, medications, and test results) were collected through in-person interviews by the principal investigator (PI), followed by a physical examination and medical record review. Furthermore, the validated Brazilian versions of the Dutch Fatigue Scale (DUFS) and the Dutch Exertion Fatigue Scale (DEFS)^(11)^ were used to assess NDs FA due to their abstract nature. These tools contain eight and nine items, respectively. Each item is followed by a 5-point Likert scale. The total score for each scale is given by the sum of the scores for each item. The total DUFS and DEFS scores were used to determine the presence of FA. The presence of FA was diagnosed when the total DUFS scores were greater than or equal to 14.5, and the presence of exertional FA was diagnosed when the total DEFS scores were greater than or equal to 12.5^(12)^.

To determine the presence of other NDs, the PI used her clinical expertise and the NANDA-I 2018-2020 classification (current at the time of data collection). These supported clinical judgment that culminated in the clinical decision-making about the presence of NDs. The data collection instruments for each ND studied considered all DCs and other elements such as definition, associated factors, associated conditions and populations at risk, as appropriate. Finally, to determine the presence of “Deficient Heart Function Syndrome” for this study, at least two NDs had to be diagnosed. Data collection lasted an average of 30 minutes. Patients were assessed by the PI at a single time point within the first six hours of admission to the ND due to expected changes in clinical status secondary to HF treatment.

Data collection was carried out only by the PI due to challenges related to the availability of professionals to collect information at the time the study was carried out, which coincided with the pandemic period.

### Data and statistics analysis

Descriptive statistics were used to describe the variables. For the proportions of categorical variables, 95% Confidence Intervals were calculated.

In order to identify a representative structure for each syndrome ND component, five models based on LCA with random effects were adjusted. LCA is an analytical technique used to calculate accuracy measures of clinical indicators when there is no perfect reference standard, considering the hypothesis that an unobserved or latent variable (ND) indicates the associations between observable variables (clinical indicators)^(9)^.

Each model was based on two latent classes to represent the presence or absence of each ND under study. Each model was considered adjusted when all the characteristics included presented at least one accuracy measure (sensitivity or specificity) with values greater than 50%, based on the lower range of their confidence interval. Then, for each adjusted model, the posterior probabilities of latent classes were calculated in order to represent the diagnostic inference to be used to define each participant’s status in relation to the presence/absence of each of the five diagnoses. After this inference, a new LCA was adjusted based on the distribution of the five diagnoses, and measures of diagnostic accuracy were obtained, which were interpreted in relation to the probability of expression of “Deficient Heart Function Syndrome”. Subsequent probabilities were also calculated to identify the different forms of expression of this syndrome among individuals assessed. For each individual diagnosis and for the syndrome, estimates of its prevalence are also presented. Finally, to analyze the simultaneous occurrence of NDs and characterize this group of diagnoses as a syndrome, log-linear models were adjusted for all possible combinations of occurrence of the five diagnoses. The model coefficients with their respective confidence intervals are presented and represent the magnitude of conditional dependence whenever their values are positive. Moreover, a Z-test was calculated to test the null hypothesis of conditional independence. A significance level of 5% was adopted for this analysis.

## RESULTS

A total of 360 individuals admitted to the ER with decompensated HF were assessed. The majority were male (n=219; 60.8%), aged between 61 and 70 years (n=102; 28.3%), white (n=213; 60.3%), retired (n=167; 46.4%) and with an average of 7.51 years of education. The majority of participants were classified as IV FC (n=187=51.9%), with hemodynamic profile B (n=229; 63.6%), reduced ejection fraction (n=257; 71.4%) and biventricular dysfunction (n=187; 51.9%). The most prevalent etiology was ischemic (n=112; 31.1%), and the major precipitants of decompensation were inadequate diet (n=126; 35%), non-adherence to medication (n=102; 28.3%) and infections (n=98; 26.4%).

FA was diagnosed in 84.17% of the sample. Seven of the 16 DCs had sensitivity and specificity greater than 80% (Table 1). The posterior probabilities for the presence of FA were strongly influenced by the simultaneous occurrence of the seven DCs that had high sensitivity and specificity.

**Table 1:** Diagnostic accuracy measures of the defining characteristics of “Fatigue”, “Excessive fluid volume”, “Decreased activity tolerance”, “Impaired comfort” and “Decreased cardiac output” nursing diagnoses, Brazil, 2024

Defining characteristics	Se	95%CI		Sp	95%CI	
“Fatigue” nursing diagnoses						
Increased rest requirement	0.9967	0.9899	1.0000	0.9649	0.9111	1.0000
Increased physical symptoms	1.0000	1.0000	1.0000	1.0000	0.9999	1.0000
Tiredness	1.0000	1.0000	1.0000	1.0000	0.9999	1.0000
Difficulty maintaining usual routines	0.9934	0.9831	1.0000	1.0000	0.9999	1.0000
Expresses lack of energy	0.9703	0.9495	0.9900	0.9824	0.9434	1.0000
Nonrestorative sleep-wake cycle	0.9175	0.8864	0.9467	0.9649	0.9122	1.0000
“Excessive fluid volume” nursing diagnosis						
Altered urine specific gravity	0.0035	0.0000	0.0109	1.0000	1.0000	1.0000
Altered blood pressure	0.5678	0.5149	0.6238	0.8594	0.7725	0.9428
Altered pulmonary artery pressure	0.8977	0.8616	0.9318	0.4843	0.3680	0.6069
Altered mental status	0.0069	0.0000	0.0177	1.0000	1.0000	1.0000
Altered respiratory pattern	0.9530	0.9262	0.9733	0.3854	0.2732	0.5097
Anasarca	0.1800	0.1379	0.2249	1.0000	0.9999	1.0000
Anxiety	0.5422	0.4866	0.5970	0.8261	0.7298	0.9174
Azotemia	0.7813	0.7346	0.8304	0.7144	0.6000	0.8160
Pulmonary congestion	0.7736	0.7204	0.8201	0.7532	0.6538	0.8464
Pleural effusion	0.1623	0.1214	0.2059	0.8859	0.8103	0.9535
Electrolyte imbalance	0.6813	0.6272	0.7388	0.7864	0.6863	0.8837
Dyspnea	0.9965	0.9893	1.0000	0.2952	0.1932	0.4050
Paroxysmal nocturnal dyspnea	0.9724	0.9512	0.9895	0.5907	0.4610	0.7100
Jugular vein distension	0.9593	0.9364	0.9806	0.6219	0.5086	0.7358
Edema	0.9385	0.9107	0.9658	0.6357	0.5277	0.7468
Weight gain over short period of time	0.6629	0.6100	0.7150	0.9504	0.8888	1.0000
Decreased serum hematocrit levels	0.5120	0.4561	0.5703	0.8018	0.7035	0.8948
Decreased serum hemoglobin	0.5155	0.4564	0.5713	0.8018	0.7019	0.8933
Hepatomegaly	0.4154	0.3650	0.4735	1.0000	0.9988	1.0000
Intake exceeds output	0.4604	0.4041	0.5202	1.0000	0.9998	1.0000
Psychomotor agitation	0.5355	0.4737	0.5935	0.8692	0.7819	0.9415
Oliguria	0.4516	0.3965	0.5103	0.9640	0.9142	1.0000
Orthopnea	0.9864	0.9724	0.9966	0.3242	0.2115	0.4296
Presence of S3 heart sound	0.2301	0.1789	0.2790	0.9501	0.8923	1.0000
Positive hepatojugular reflex	0.8500	0.8061	0.8886	0.8528	0.7643	0.9280
Adventitious respiratory sounds	0.9119	0.8767	0.9421	0.5420	0.4229	0.6603
“Decreased activity tolerance” nursing diagnosis						
Electrocardiogram change	0.9026	0.8672	0.9999	0.3879	0.3122	0.4620
Expresses fatigue	0.8462	0.7891	0.9000	0.1515	0.0984	0.2086
Generalized weakness	0.9641	0.9372	0.9920	0.0667	0.0300	0.1091
Abnormal heart rate response to activity	0.9999	0.9999	0.9999	0.9997	0.7543	0.9998
Abnormal blood pressure response to activity	0.1488	0.1039	0.2214	1.0000	1.0000	1.0000
“Impaired comfort” nursing diagnosis						
Reports altered sleep-wake cycle	0.9193	0.8728	0.9602	0.1156	0.0727	0.1569
Anxiety	0.9936	0.9789	1.0000	0.9494	0.9179	0.9788
Crying	0.0682	0.0317	0.1090	0.9898	0.9729	1.0000
Uneasy in situation	1.0000	1.0000	1.0000	0.0050	0.0000	0.0157
Expresses discontentment with situation	0.9939	0.9805	1.0000	0.0152	0.0000	0.0329
Difficulty relaxing	0.9064	0.8563	0.9517	0.9895	0.9708	1.0000
Psychomotor agitation	0.9869	0.9630	1.0000	0.9893	0.9719	1.0000
Irritable mood	0.2485	0.1798	0.3202	1.0000	0.9999	1.0000
Moaning	0.0251	0.0059	0.0531	0.9901	0.9746	1.0000
Expresses fear	0.1863	0.1278	0.2483	0.9246	0.8859	0.9592
Itching	0.0124	0.0000	0.0318	1.0000	1.0000	1.0000
Expresses feeling warm	0.0062	0.0000	0.0198	0.9899	0.9742	1.0000
Expresses discomfort	0.9753	0.9483	0.9941	0.0655	0.0359	0.1012
Reports hunger	0.0000	0.0000	0.0000	0.9950	0.9838	1.0000
Expresses feeling cold	0.0559	0.0195	0.0928	0.9497	0.9147	0.9792
Symptoms of distress	0.0000	0.0000	0.0000	0.9899	0.9737	1.0000
“Decreased cardiac output” nursing diagnosis						
Electrocardiogram change	0.7836	0.7103	0.8552	1.0000	1.0000	1.0000
Bradycardia	0.0523	0.0177	0.0938	1.0000	1.0000	1.0000
Tachycardia	0.4179	0.3333	0.4966	1.0000	1.0000	1.0000
Heart palpitations	0.4179	0.3333	0.5000	1.0000	1.0000	1.0000
Jugular vein distension	0.7537	0.6791	0.8294	1.0000	1.0000	1.0000
Edema	0.7463	0.6739	0.8306	1.0000	1.0000	1.0000
Fatigue	0.9403	0.8925	0.9762	1.0000	1.0000	1.0000
Weight gain	0.5746	0.4921	0.6538	1.0000	1.0000	1.0000
Heart murmur	0.2463	0.1711	0.3233	1.0000	1.0000	1.0000
Altered blood pressure	0.9328	0.8881	0.9724	1.0000	1.0000	1.0000
Abnormal skin color	0.9030	0.8473	0.9510	1.0000	1.0000	1.0000
Dyspnea	0.9701	0.9360	0.9932	1.0000	1.0000	1.0000
Oliguria	0.6940	0.6115	0.7724	1.0000	1.0000	1.0000
Clammy skin	0.4179	0.3333	0.4960	1.0000	1.0000	1.0000
Decreased peripheral pulses	0.8955	0.8361	0.9453	1.0000	1.0000	1.0000
Prolonged capillary refill	0.9701	0.9384	0.9930	1.0000	1.0000	1.0000
Paroxysmal nocturnal dyspnea	0.7910	0.7222	0.8547	0.9911	0.9769	1.0000
Decreased ejection fraction	0.8880	0.8308	0.9396	1.0000	1.0000	1.0000
Orthopnea	0.8806	0.8245	0.9369	1.0000	1.0000	1.0000
Presence of S3 heart sound	0.3209	0.2394	0.4030	1.0000	1.0000	1.0000
Presence of S4 heart sound	0.0299	0.0071	0.0602	1.0000	1.0000	1.0000
Adventitious respiratory sounds	0.7612	0.6803	0.8346	1.0000	1.0000	1.0000
Cough	0.2164	0.1515	0.2846	1.0000	1.0000	1.0000
Anxiety	0.5672	0.4825	0.6529	1.0000	1.0000	1.0000
Psychomotor agitation	0.5746	0.4863	0.6591	1.0000	1.0000	1.0000

*Se - sensitivity; Sp - specificity; 95%CI - 95% Confidence Interval.*

EFV was diagnosed in 80.24% of the sample. All of its DCs, except “Increased central venous pressure”, were identified and presented sensitivity and specificity measures higher than 50%. Of this set, eight DCs showed high sensitivity (>80%); 15 showed high specificity (>70%); and three showed similar sensitivity and specificity (>70%) (Table 1). The DCs with the highest sensitivity were present in almost all combinations that demonstrated a higher probability for the presence of diagnosis.

The prevalence of DAT was 54.16%. All of its DCs were identified in the sample. However, two of them (“Exertional discomfort” and “Exertional dyspnea”) were identified in all participants and did not compose the final LCA. Of the remaining five, three showed high sensitivity (>70%); one showed high specificity (100%); and another presented high values for both sensitivity and specificity (>70%) (Table 1). The posterior probabilities showed the importance of the “Abnormal heart rate response to activity” DC, as it comprises all the combinations that point to a greater probability of this diagnosis occurring.

The estimated prevalence of IC was 44.71%. Nine DCs showed high specificity (>90%). On the other hand, four DCs showed high sensitivity (>80%), and three DCs showed high values of both sensitivity and specificity (>90%) (Table 1). All sets consisted of at least two DCs with high sensitivity and one with high specificity.

The prevalence of DCO was 37.22%. The low prevalence was due to the absence of DCs of this ND in the sample. Unlike the other NDs, 11 DCs were not identified in the sample, and the remaining 25 had higher specificity. Furthermore, 14 DCs also had sensitivity greater than 50%; eight had sensitivity greater than 80%; and two had sensitivity greater than 90% (Table 1). LCA revealed a large number of different combinations of several DCs occurring concomitantly.

Table 1 presents the diagnostic accuracy measures of the DCs of the studied NDs.

After using the posterior probabilities of each ND to determine the presence or absence of each ND, the syndrome’s LCA included the five diagnoses. Table 2 presents the accuracy measures of the “Deficient Heart Function Syndrome” DC and its prevalence. In this model, EFV and FA had the highest sensitivity values, while DCO and IC had the highest specificity. Both sensitivity and specificity for DAT were approximately 70%.

**Table 2:** Diagnostic accuracy measures of the defining characteristics of “Deficient Heart Function Syndrome” in people with decompensated heart failure, Brazil, 2024

Defining characteristics	Se	95%CI		Sp	95%CI	
Excessive fluid volume	0.9072	0.8666	0.9870	0.3567	0.2487	0.5204
Decreased cardiac output	0.4357	0.3631	0.5371	0.7292	0.6235	0.8344
Decreased activity tolerance	0.6806	0.6115	0.7973	0.6803	0.5397	0.8527
Fatigue	0.8352	0.7756	0.8966	0.1481	0.0736	0.2235
Impaired comfort	0.7317	0.5803	0.9988	0.9998	0.8134	0.9999
Estimated prevalence of the alleged syndrome: 61.49%						

*Se - sensitivity; Sp - specificity; 95%CI - 95% Confidence Interval.*

Table 3 shows the posterior probabilities for the occurrence of “Deficient Heart Function Syndrome”.

**Table 3:** Posterior probabilities for the occurrence of “Deficient Heart Function Syndrome” in people with decompensated heart failure according to different sets of defining characteristics, Brazil, 2024

Sets	Defining characteristics					n	Syndrome posterior probability	
	EFV	DCO	DAT	FA	IC		Present	Absent
2	No	No	No	No	Yes	1	1.00	0.00
5	No	No	Yes	No	Yes	5	1.00	0.00
7	No	No	Yes	Yes	Yes	3	1.00	0.00
9	No	Yes	No	No	Yes	1	1.00	0.00
11	No	Yes	No	Yes	Yes	1	1.00	0.00
13	No	Yes	Yes	No	Yes	1	1.00	0.00
15	No	Yes	Yes	Yes	Yes	3	1.00	0.00
17	Yes	No	No	No	Yes	5	1.00	0.00
19	Yes	No	No	Yes	Yes	23	1.00	0.00
21	Yes	No	Yes	No	Yes	7	1.00	0.00
23	Yes	No	Yes	Yes	Yes	45	1.00	0.00
25	Yes	Yes	No	No	Yes	2	1.00	0.00
27	Yes	Yes	No	Yes	Yes	20	1.00	0.00
29	Yes	Yes	Yes	No	Yes	7	1.00	0.00
30	Yes	Yes	Yes	Yes	No	20	0.67	0.33
31	Yes	Yes	Yes	Yes	Yes	38	1.00	0.00

*EFV - excessive fluid volume; DCO - decreased cardiac output; DAT - decreased activity tolerance; FA - fatigue; IC - impaired comfort; n - frequency of each combination of the five nursing diagnoses.*

In Table 3, the sets represent the different combinations (present/absent) of each ND (in the case of the syndrome, DCs would be the five NDs investigated). These sets were obtained directly from the sample, and their frequency is described in the “n” column. A total of 31 different combinations were identified. In the table, only the combinations in which the syndrome presence probability was greater than 0.5 were shown. Furthermore, the posterior probabilities suggest a strong influence of IC, since this ND was present in almost all sets that demonstrated a higher probability of the syndrome occurring. Most of participants identified with the syndrome presented three or more NDs concomitantly.

Table 4 presents the log-linear models that represent the combinations of the five diagnoses that exhibited conditional dependence relationships between them.

**Table 4:** Log-linear models including different statuses of nursing diagnoses that make up “Deficient Heart Function Syndrome” in people with decompensated heart failure, Brazil, 2024

Parameter	Coef.	SE	Z	*p* value	IC95%	
Constant	1.70	0.43	4.00	<0.001	0.87	2.54
[EFV = P] ^*^ [DCO = P] ^*^ [DAT = P] ^*^ [FA = P] ^*^ [IC = P]	1.95	0.46	4.27	<0.001	1.05	2.84
[EFV = P] ^*^ [DCO = P] ^*^ [DAT = P] ^*^ [FA = P] ^*^ [IC = A]	1.32	0.48	2.74	0.006	0.37	2.26
[EFV = P] ^*^ [DCO = P] ^*^ [DAT = A] ^*^ [FA = P] ^*^ [IC = P]	1.32	0.48	2.74	0.006	0.37	2.26
[EFV = P] ^*^ [DCO = A] ^*^ [DAT = P] ^*^ [FA = P] ^*^ [IC = P]	2.11	0.45	4.68	<0.001	1.23	3.00
[EFV = P] ^*^ [DCO = P] ^*^ [DAT = A] ^*^ [FA = P] ^*^ [IC = A]	1.10	0.49	2.23	0.026	0.13	2.06
[EFV = P] ^*^ [DCO = A] ^*^ [DAT = P] ^*^ [FA = P] ^*^ [IC = A]	2.04	0.45	4.51	<0.001	1.16	2.93
[EFV = P] ^*^ [DCO = A] ^*^ [DAT = A] ^*^ [FA = P] ^*^ [IC = P]	1.45	0.47	3.07	0.002	0.52	2.38
[EFV = P] ^*^ [DCO = A] ^*^ [DAT = A] ^*^ [FA = P] ^*^ [IC = A]	2.20	0.45	4.89	<0.001	1.32	3.08
[EFV = A] ^*^ [DCO = A] ^*^ [DAT = A] ^*^ [FA = P] ^*^ [IC = A]	1.27	0.48	2.62	0.009	0.32	2.21

*EFV - excessive fluid volume; DCO - decreased cardiac output; DAT - decreased activity tolerance; FA - fatigue; IC - impaired comfort; P - present; A - absent; Coef - coefficient; SE - standard error; Z - Z test; 95%CI - 95% Confidence Interval.*

As shown in Table 4, nine combinations of the five NDs showed conditional dependence. The concomitant presence of the five NDs indicates a strong relationship between them.

## DISCUSSION

This is the first study to examine whether FA, EFV, DAT, IC, and DCO constitute “Deficient Heart Function Syndrome”.

In line with our findings, FA was highly prevalent in HF in another investigation^(5)^. This is consistent with the pathophysiological mechanisms of HF of water retention and poor tissue perfusion, which results in a sustained perception of tiredness and inability to perform usual physical and mental activities. However, due to the subjective nature of this ND, its identification requires specific training, in addition to the use of reliable assessment methods^(13)^.

EFV was present in 80.24% of participants. The DCs with the highest sensitivity portray classic manifestations presented by people with decompensated HF^(14,15)^. This myriad of signs and symptoms reflects volume overload^(1)^. In fact, this ND is frequently documented in the presence of congestive symptoms^(16)^, and is related to the risk of clinical worsening^(17)^.

The prevalence of IC was 44.71% in our study, which contrasts with a prevalence of 86% found in a retrospective study of 144 medical records of older adults with HF^(18)^. This difference may be due to the time period in which data were collected from participants. Meanwhile, in our study, we included participants who had been admitted to the ND for no more than six hours, and in the aforementioned study, the researchers took into account the entire length of hospital stay. Physical comfort is affected in people with decompensated HF due to the worsening of clinical manifestations that negatively impact daily activities and quality of life^(6)^.

The prevalence of DAT was 54.16% in our sample. The literature is prolific in demonstrating that this ND is common in people with decompensated HF^(5,13)^. Three DCs of DAT that were considered highly sensitive in this study were critical in providing clinical evidence to support this ND in a previous study^(19)^. Among these DCs, “Abnormal heart rate response to activity” appears to be of particular relevance and is attributed to DCO^(20)^.

DCO had the lowest prevalence among the five NDs. Regarding the DCs with the highest sensitivity, a Brazilian survey of people with HF identified five DCs for this diagnosis, supporting the findings of this study^(13)^. The DCs with the highest specificity suggest the critical and priority nature of this diagnosis for this population^(14)^. However, DCO was not identified in most participants in this investigation due to the phenotypic variation of decompensated HF, since some participants did not present signs of poor perfusion, a vital element for the typification of this diagnosis^(15)^. Similar to our findings, other authors identified DCO in 16% of their sample^(18)^. In another recent study, four new related factors were found for DCO, supporting its relevance in clinical practice^(21)^.

This study provides evidence that the five NDs (FA, EFV, DAT, IC, and DCO) occur simultaneously, suggesting that together they constitute “Deficient Heart Function Syndrome”. This evidence is supported by the strong relationship between the NDs and their conditional dependence. In fact, HF presents a multifaceted and cascading behavior, portraying the influence of one event on the appearance of another^(22)^. Likewise, an affected human response influences the expression of other human responses, which may signal the relevance of a syndrome diagnosis.

The estimated prevalence of “Deficient Heart Function Syndrome” was 61.49%. EFV and FA had the highest sensitivity, which refers to the ability of these NDs to identify individuals with the syndrome. Since they are highly sensitive NDs, only a few individuals diagnosed with the syndrome will be missed and the number of false negative results is low^(23)^. In turn, DCO and IC had the highest specificity. This means that if these NDs are not identified, it is likely that individuals do not have the syndrome ND. Since DCO and IC are highly specific, when identified, there will be few false-positive cases of the syndrome.

It is worth mentioning that the NANDA-I^(24)^ 2024-2026 edition excluded DCO and IC from its structure, but even so, these NDs have been the most frequently used by nurses^(5)^, having been observed, through this study, a significant prevalence of each of them based on clinical evidence and their occurrence together in the clinical context in which it was applied.

### Study limitations

The generalizability of our findings is subject to certain limitations, and cannot be extrapolated to all patients with HF, since the syndrome ND was investigated only in people with decompensated HF and in a single highly specialized center. Furthermore, the fact that assessments were performed exclusively by the PI, without validity by other specialists, may be a bias, despite the PI’s efforts to accurately document clinical findings and decision-making. Furthermore, data collection occurred during the pandemic, which may have influenced the findings on FA. Finally, elements from the previous edition of NANDA-I (2018-2020), a publication valid at the time the study was conducted, were considered.

### Contributions to nursing

For the first time in the literature, it has been demonstrated that FA, EFV, DAT, HF, and DCO can constitute the “Deficient Heart Function Syndrome” ND. Addressing human responses as a syndrome has several advantages: the syndrome may more accurately represent the human response to HF; managing a syndrome ND may be more cost-effective than addressing each ND separately; and a syndrome ND will facilitate appropriate nursing documentation. Regarding research, future studies should investigate clinical validity of “Deficient Heart Function Syndrome” in other groups of people. In addition, studies to map nursing-sensitive outcomes for this ND and intervention tests should be performed. Finally, this study made it possible to identify that the NDs that constitute the syndromic diagnosis are identified in clinical practice, adding evidence of the need for new investigations that support the new inclusion of NDs removed from the current edition of NANDA-I.

## CONCLUSIONS

The simultaneous occurrence of FA, EFV, DAT, IC and DCO indicates a strong relationship between them, providing strong evidence of a syndrome ND in people with decompensated HF. Furthermore, the occurrence of these NDs in clusters as an unfolding of common causal factors can be addressed with similar nursing interventions.

## Data Availability

The research data are available within the article.
