# Supplementary material for: Deficient Heart Function Syndrome: a new nursing diagnosis for people with heart failure
Source: Rev Bras Enferm. 2025 Oct 3;78(4):e20240653. doi: 10.1590/0034-7167-2024-0653 (PMC12494352; doi:10.1590/0034-7167-2024-0653)
Supplement: Translation

# Translation: Síndrome da Função Cardíaca Deficiente: novo diagnóstico de enfermagem para pessoas com insuficiência cardíaca

**DOI:** 10.1590/0034-7167-2024-0653pt

**Authors:** Ana Paula Dias de Oliveira, Marcos Venícios de Oliveira Lopes, Rita de Cassia Gengo e Silva Butcher, Viviane Martins da Silva, Vinicius Batista Santos, Juliana de Lima Lopes, Alba Lucia Bottura Leite de Barros

**Affiliations:** I Universidade Federal de São Paulo. São Paulo, São Paulo, Brasil; II Universidade Federal do Ceará. Fortaleza, Ceará, Brasil; III Connell School of Nursing. Chestnut, Estado Unidos da América

**Keywords:** Síndrome, Insuficiência Cardíaca, Diagnóstico de Enfermagem, Processo de Enfermagem, Terminologia Padronizada em Enfermagem.

## Abstract

**Objetivos:**

identificar se o conjunto de diagnósticos de enfermagem “Volume de líquido excessivo”, “Débito cardíaco diminuído”, “Tolerância à atividade diminuída”, “Fadiga” e “Conforto prejudicado” constitui a “Síndrome da Função Cardíaca Deficiente” em pessoas com insuficiência cardíaca.

**Métodos:**

estudo transversal analítico que incluiu 360 participantes que foram avaliados por enfermeira especialista em cardiologia que utilizou o processo de enfermagem e classificação da NANDA-Internacional. Análise de classes latentes e modelos log-lineares foram utilizados para determinar a ocorrência de diagnósticos de enfermagem por cluster.

**Resultados:**

“Fadiga” e “Volume de líquido excessivo” apresentaram maior sensibilidade, “Débito cardíaco diminuído” e “Conforto prejudicado” apresentaram maior especificidade. “Tolerância à atividade diminuída” apresentou sensibilidade e especificidade semelhantes. Análise de classe latente para o novo diagnóstico incluiu todos os diagnósticos. Nove combinações dos status dos diagnósticos exibiram dependência condicional.

**Conclusões:**

a presença concomitante e a forte relação entre os diagnósticos fornecem evidências da existência do diagnóstico sindrômico nessa população.

## INTRODUÇÃO

A insuficiência cardíaca (IC) é uma condição de saúde altamente prevalente e debilitante^(1)^ que envolve processos patológicos complexos que afetam várias dimensões da vida de uma pessoa^(2)^. Como resultado, indivíduos com IC experimentam múltiplas respostas humanas simultaneamente^(3)^, representadas por *clusters* de diagnósticos de enfermagem (DEs). Esses *clusters* são caracterizados como uma síndrome se compreenderem pelo menos dois diagnósticos aprovados pela NANDA-*International* (NANDA-I) e as mesmas intervenções forem usadas para tratá-los^(4)^.

De acordo com revisão da literatura, pessoas com IC geralmente experimentam vários DEs simultaneamente, sendo frequentemente diagnosticados conjuntamente pelos enfermeiros na prática clínica, e por essa razão, foram selecionados para compor o diagnóstico sindrômico do presente estudo. Esses DEs incluem “Fadiga” (FA), “Volume de líquido excessivo” (VLE), “Tolerância à atividade diminuída” (TAD) e “Débito cardíaco diminuído” (DCD)^(5)^. A compreensão ampliada do termo “síndrome” como condição multidimensional influenciou a inclusão do DE “Conforto prejudicado” (CP), com a finalidade de atender ao anseio de compor um DE que identificasse elementos emocionais e sociais comprometidos, além dos físicos.

O VLE está inserido no domínio 2 (nutrição) e classe 5 (hidratação); DCD, TAD e FA estão inseridos no domínio 4 (atividade/repouso), sendo os dois primeiros diagnósticos na classe 4 (respostas cardiovasculares/pulmonares), e o terceiro, na classe 3 (equilíbrio de energia). Por fim, o CP está incorporado no domínio 12 (conforto) e classe 1 (conforto físico).

Acredita-se que a ocorrência desses DEs como um *cluster* seja o resultado de um processo fisiopatológico comum (função cardíaca deficiente) que pode ser abordado com intervenções semelhantes. Assim, alterações na função do músculo cardíaco resultam na ativação de respostas adaptativas que desencadeiam má perfusão dos tecidos^(1)^ (correlacionado com DCD) e sobrecarga volêmica^(1)^ (correlacionado com VLE). Além disso, hipoperfusão culmina na percepção sustentada de energia insuficiente para a realização de atividades diárias^(1)^ (correlacionado com TAD) e sensação incapacitante de exaustão física e mental^(1)^ (correlacionado com FA). Somado ao desconforto físico, sentimentos negativos advêm de hospitalizações frequentes, dependência de cuidadores e interrupção de atividades laborais^(6)^ (correlacionado com CP). Ainda assim, nenhum dos DEs de síndrome aprovados na classificação (2018-2020)^(4)^, edição vigente, por ocasião do início do estudo, parecia adequada para documentar com precisão as respostas humanas apresentadas por pessoas com IC descompensada.

Portanto, o DE “Síndrome da Função Cardíaca Deficiente” foi desenvolvido com base na literatura científica em saúde e na experiência clínica dos autores deste estudo. Foi definido como “sofrimento físico, emocional, psicossocial e espiritual devido a uma diminuição da capacidade do coração de bombear sangue para o corpo e às respostas adaptativas ao músculo cardíaco insultado”. As características definidoras (CDs) do DE sindrômico são FA, VLE, TAD, CP e DCD. O fator relacionado compreende a disfunção cardíaca estrutural e/ou funcional.

## OBJETIVOS

Identificar se o conjunto de DEs FA, VLE, TAD, CP e DCD constitui a “Síndrome da Função Cardíaca Deficiente” em pessoas com IC.

## MÉTODOS

### Aspectos éticos

Este estudo foi conduzido de acordo com os princípios éticos, e foi aprovado pelo Comitê de Ética em Pesquisa. O Termo de Consentimento Livre e Esclarecido (TCLE) foi obtido de todos os indivíduos envolvidos por meio escrito, e a confidencialidade dos dados foi assegurada^(7)^.

### Desenho, período e local do estudo

Trata-se de estudo transversal analítico que seguiu as diretrizes do *STrengthening the Reporting of OBservational studies in Epidemiology* ^(8)^. A coleta de dados foi realizada entre abril de 2019 e maio de 2022, em um pronto-socorro (PS) público e especializado em condições cardiovasculares, em São Paulo, Brasil.

### População ou amostra; critérios de inclusão e exclusão

O tamanho da amostra foi estabelecido considerando a técnica de análise de classes latentes (ACL) para inferência de cada DE individualmente^(9)^. O DCD foi selecionado para definir o tamanho da amostra, pois possui o maior número de CD (n=36)^(4)^. Uma proporção de dez participantes por CD determinou o tamanho da amostra em 360.

Foram incluídos participantes com diagnóstico médico de IC descompensada, com idade igual ou superior a 18 anos, internados no PS por não mais de seis horas e com classes funcionais (CFs) III e IV da *New York Heart Association* ^(10)^. Foram excluídos os participantes com rebaixamento do nível de consciência e/ou confusão, instabilidade hemodinâmica e que necessitaram de intubação traqueal durante a coleta de dados.

### Protocolo do estudo

Todas as pessoas admitidas no PS com IC descompensada que atenderam aos critérios de elegibilidade foram convidados a participar do estudo e assinaram o TCLE.

Dados sociodemográficos (idade, sexo, raça, estado civil, religião, escolaridade e ocupação) e clínicos (história de saúde pregressa e atual, medicamentos e resultados de exames) foram coletados por meio de entrevistas presenciais pela investigadora principal (IP), seguidos da realização de exame físico e revisão de prontuário. Além disso, as versões brasileiras validadas da *Dutch Fatigue Scale* (DUFS) e da *Dutch Exertion Fatigue Scale* (DEFS)^(11)^ foram utilizadas para avaliar o DE FA em virtude de seu caráter abstrato. As referidas ferramentas contêm oito e nove itens, respectivamente. Cada item é seguido por uma escala Likert de 5 pontos. A pontuação total de cada escala é dada pela soma das pontuações de cada item. Os escores totais da DUFS e da DEFS foram utilizados para determinar a presença de FA. A presença de FA foi diagnosticada quando os escores totais da DUFS foram maiores ou iguais a 14,5, e a presença de FA por esforço foi diagnosticada quando os escores totais da DEFS foram maiores ou iguais a 12,5^(12)^.

Para determinar a presença dos demais DEs, a IP valeu-se de sua *expertise* clínica e da classificação NANDA-I 2018-2020 (atual no momento da coleta de dados). Esses apoiaram o julgamento clínico que culminou na tomada de decisão clínica sobre a presença dos DEs. Os instrumentos de coleta de dados de cada DE estudado consideraram todas as CDs e demais elementos como definição, fatores associados, condições associadas e populações em risco, conforme apropriado. Por fim, para determinar a presença de “Síndrome da Função Cardíaca Deficiente” para este estudo, pelo menos dois DEs deveriam ter sido diagnosticados. A coleta de dados durou em média 30 minutos. Os pacientes foram avaliados pela IP em um único momento nas primeiras seis horas da admissão no PS em razão das mudanças esperadas no quadro clínico secundário ao tratamento da IC.

A coleta de dados foi realizada apenas pela IP devido aos desafios relacionados à disponibilidade de profissionais para recolha de informações no momento de realização do estudo, que coincidiu com o período pandêmico.

### Análise dos dados e estatística

Estatística descritiva foi utilizada para descrever as variáveis. Para as proporções das variáveis categóricas, foram calculados Intervalos de Confiança de 95%.

A fim de identificar uma estrutura representativa para cada componente do DE da síndrome, foram ajustados cinco modelos baseados em ACL com efeitos aleatórios. A ACL é uma técnica analítica utilizada para calcular medidas de acurácia de indicadores clínicos quando não existe um padrão de referência perfeito, considerando a hipótese de que uma variável (DE) não observada ou latente indica as associações entre variáveis observáveis (indicadores clínicos)^(9)^.

Cada modelo foi baseado em duas classes latentes para representar a presença ou ausência de cada DE em estudo. Cada modelo foi considerado ajustado quando todas as características incluídas apresentaram pelo menos uma medida de acurácia (sensibilidade ou especificidade) com valores superiores a 50%, com base na faixa inferior de seu intervalo de confiança. Em seguida, para cada modelo ajustado, foram calculadas as probabilidades posteriores das classes latentes, a fim de representar a inferência diagnóstica a ser utilizada para definir o *status* de cada participante em relação à presença/ausência de cada um dos cinco diagnósticos. Após essa inferência, uma nova ACL foi ajustada com base na distribuição dos cinco diagnósticos, e foram obtidas medidas de acurácia diagnóstica, que foram interpretadas em relação à probabilidade de expressão da “Síndrome da Função Cardíaca Deficiente”. Probabilidades subsequentes também foram calculadas para identificar as diferentes formas de expressão dessa síndrome entre as pessoas avaliadas. Para cada diagnóstico individual e para a síndrome, também são apresentadas estimativas de sua prevalência. Por fim, para analisar a ocorrência simultânea dos DEs e caracterizar esse grupo de diagnósticos como uma síndrome, foram ajustados modelos log-lineares para todas as combinações possíveis de ocorrência dos cinco diagnósticos. Os coeficientes do modelo com respectivos Intervalos de Confiança são apresentados e representam a magnitude da dependência condicional sempre que seus valores são positivos. Além disso, um teste Z foi calculado para testar a hipótese nula de independência condicional. Adotou-se um nível de significância de 5% para esta análise.

## RESULTADOS

Foram avaliadas 360 pessoas admitidas no PS com IC descompensada. A maioria era do sexo masculino (n=219; 60,8%), com idade entre 61 e 70 anos (n=102; 28,3%), branca (n=213; 60,3%), aposentada (n=167; 46,4%) e com média de 7,51 anos de estudo. A maioria dos participantes foi classificada na CF IV (n=187=51,9%), com perfil hemodinâmico B (n=229; 63,6%), fração de ejeção reduzida (n=257; 71,4%) e disfunção biventricular (n=187; 51,9%). A etiologia mais prevalente foi a isquêmica (n=112; 31,1%), e os maiores precipitadores da descompensação foram dieta inadequada (n=126; 35%), não adesão medicamentosa (n=102; 28,3%) e infecções (n=98; 26,4%).

A FA foi diagnosticada em 84,17% da amostra. Sete das 16 CDs apresentaram sensibilidade e especificidade superiores a 80% (Tabela 1). As probabilidades posteriores para a presença de FA foram fortemente influenciadas pela ocorrência simultânea das sete CDs que apresentaram alta sensibilidade e especificidade.

**Tabela 1:** Medidas de precisão diagnósticas das características definidoras dos diagnósticos de enfermagem “Fadiga”, “Volume de líquido excessivo”, “Tolerância à atividade diminuída”, “Conforto prejudicado” e “Débito cardíaco diminuído”, Brasil, 2024

Características definidoras	Se	IC95%		Esp	IC95%	
Diagnóstico de enfermagem “Fadiga”						
Aumento da necessidade de descanso	0,9967	0,9899	1,0000	0,9649	0,9111	1,0000
Aumento dos sintomas físicos	1,0000	1,0000	1,0000	1,0000	0,9999	1,0000
Cansaço	1,0000	1,0000	1,0000	1,0000	0,9999	1,0000
Incapacidade para manter a rotina diária	0,9934	0,9831	1,0000	1,0000	0,9999	1,0000
Energia insuficiente	0,9703	0,9495	0,9900	0,9824	0,9434	1,0000
Padrão de sono não restaurador	0,9175	0,8864	0,9467	0,9649	0,9122	1,0000
Diagnóstico de enfermagem “Volume de líquido excessivo”						
Alteração na gravidade da urina	0,0035	0,0000	0,0109	1,0000	1,0000	1,0000
Alteração na pressão arterial	0,5678	0,5149	0,6238	0,8594	0,7725	0,9428
Alteração na pressão da artéria pulmonar	0,8977	0,8616	0,9318	0,4843	0,3680	0,6069
Alteração no estado mental	0,0069	0,0000	0,0177	1,0000	1,0000	1,0000
Alteração do padrão respiratório	0,9530	0,9262	0,9733	0,3854	0,2732	0,5097
Anasarca	0,1800	0,1379	0,2249	1,0000	0,9999	1,0000
Ansiedade	0,5422	0,4866	0,5970	0,8261	0,7298	0,9174
Azotemia	0,7813	0,7346	0,8304	0,7144	0,6000	0,8160
Congestão pulmonar	0,7736	0,7204	0,8201	0,7532	0,6538	0,8464
Derrame pleural	0,1623	0,1214	0,2059	0,8859	0,8103	0,9535
Desequilíbrio eletrolítico	0,6813	0,6272	0,7388	0,7864	0,6863	0,8837
Dispneia	0,9965	0,9893	1,0000	0,2952	0,1932	0,4050
Dispneia paroxística noturna	0,9724	0,9512	0,9895	0,5907	0,4610	0,7100
Distensão da veia jugular	0,9593	0,9364	0,9806	0,6219	0,5086	0,7358
Edema	0,9385	0,9107	0,9658	0,6357	0,5277	0,7468
Ganho de peso	0,6629	0,6100	0,7150	0,9504	0,8888	1,0000
Hematócrito diminuído	0,5120	0,4561	0,5703	0,8018	0,7035	0,8948
Hemoglobina diminuída	0,5155	0,4564	0,5713	0,8018	0,7019	0,8933
Hepatomegalia	0,4154	0,3650	0,4735	1,0000	0,9988	1,0000
Ingestão maior que a eliminação	0,4604	0,4041	0,5202	1,0000	0,9998	1,0000
Inquietação	0,5355	0,4737	0,5935	0,8692	0,7819	0,9415
Oligúria	0,4516	0,3965	0,5103	0,9640	0,9142	1,0000
Ortopneia	0,9864	0,9724	0,9966	0,3242	0,2115	0,4296
Presença de 3ª bulha cardíaca	0,2301	0,1789	0,2790	0,9501	0,8923	1,0000
Reflexo hepatojugular positivo	0,8500	0,8061	0,8886	0,8528	0,7643	0,9280
Ruídos adventícios respiratórios	0,9119	0,8767	0,9421	0,5420	0,4229	0,6603
Diagnóstico de enfermagem “Tolerância à atividade diminuída”						
Alteração no eletrocardiograma	0,9026	0,8672	0,9999	0,3879	0,3122	0,4620
Fadiga	0,8462	0,7891	0,9000	0,1515	0,0984	0,2086
Fraqueza generalizada	0,9641	0,9372	0,9920	0,0667	0,0300	0,1091
Resposta anormal da frequência cardíaca à atividade	0,9999	0,9999	0,9999	0,9997	0,7543	0,9998
Resposta anormal da pressão arterial à atividade	0,1488	0,1039	0,2214	1,0000	1,0000	1,0000
Diagnóstico de enfermagem “Conforto prejudicado”						
Alteração no padrão de sono	0,9193	0,8728	0,9602	0,1156	0,0727	0,1569
Ansiedade	0,9936	0,9789	1,0000	0,9494	0,9179	0,9788
Choro	0,0682	0,0317	0,1090	0,9898	0,9729	1,0000
Desconforto com a situação	1,0000	1,0000	1,0000	0,0050	0,0000	0,0157
Descontentamento com a situação	0,9939	0,9805	1,0000	0,0152	0,0000	0,0329
Incapacidade de relaxar	0,9064	0,8563	0,9517	0,9895	0,9708	1,0000
Inquietação	0,9869	0,9630	1,0000	0,9893	0,9719	1,0000
Irritabilidade	0,2485	0,1798	0,3202	1,0000	0,9999	1,0000
Lamento	0,0251	0,0059	0,0531	0,9901	0,9746	1,0000
Medo	0,1863	0,1278	0,2483	0,9246	0,8859	0,9592
Prurido	0,0124	0,0000	0,0318	1,0000	1,0000	1,0000
Sensação de calor	0,0062	0,0000	0,0198	0,9899	0,9742	1,0000
Sensação de desconforto	0,9753	0,9483	0,9941	0,0655	0,0359	0,1012
Sensação de fome	0,0000	0,0000	0,0000	0,9950	0,9838	1,0000
Sensação de frio	0,0559	0,0195	0,0928	0,9497	0,9147	0,9792
Sintomas de sofrimento	0,0000	0,0000	0,0000	0,9899	0,9737	1,0000
Diagnóstico de enfermagem “Débito cardíaco diminuído”						
Alteração do eletrocardiograma	0,7836	0,7103	0,8552	1,0000	1,0000	1,0000
Bradicardia	0,0523	0,0177	0,0938	1,0000	1,0000	1,0000
Taquicardia	0,4179	0,3333	0,4966	1,0000	1,0000	1,0000
Palpitações cardíacas	0,4179	0,3333	0,5000	1,0000	1,0000	1,0000
Distensão da veia jugular	0,7537	0,6791	0,8294	1,0000	1,0000	1,0000
Edema	0,7463	0,6739	0,8306	1,0000	1,0000	1,0000
Fadiga	0,9403	0,8925	0,9762	1,0000	1,0000	1,0000
Ganho de peso	0,5746	0,4921	0,6538	1,0000	1,0000	1,0000
Sopro cardíaco	0,2463	0,1711	0,3233	1,0000	1,0000	1,0000
Alteração na pressão arterial	0,9328	0,8881	0,9724	1,0000	1,0000	1,0000
Cor da pele anormal	0,9030	0,8473	0,9510	1,0000	1,0000	1,0000
Dispneia	0,9701	0,9360	0,9932	1,0000	1,0000	1,0000
Oligúria	0,6940	0,6115	0,7724	1,0000	1,0000	1,0000
Pele pegajosa	0,4179	0,3333	0,4960	1,0000	1,0000	1,0000
Pulsos periféricos diminuídos	0,8955	0,8361	0,9453	1,0000	1,0000	1,0000
Enchimento capilar prolongado	0,9701	0,9384	0,9930	1,0000	1,0000	1,0000
Dispneia paroxística noturna	0,7910	0,7222	0,8547	0,9911	0,9769	1,0000
Fração de ejeção diminuída	0,8880	0,8308	0,9396	1,0000	1,0000	1,0000
Ortopneia	0,8806	0,8245	0,9369	1,0000	1,0000	1,0000
Presença de 3ª bulha cardíaca	0,3209	0,2394	0,4030	1,0000	1,0000	1,0000
Presença da 4ª bulha cardíaca	0,0299	0,0071	0,0602	1,0000	1,0000	1,0000
Ruídos adventícios respiratórios	0,7612	0,6803	0,8346	1,0000	1,0000	1,0000
Tosse	0,2164	0,1515	0,2846	1,0000	1,0000	1,0000
Ansiedade	0,5672	0,4825	0,6529	1,0000	1,0000	1,0000
Inquietação	0,5746	0,4863	0,6591	1,0000	1,0000	1,0000

*Se - sensibilidade; Esp - especificidade; IC95% - Intervalo de Confiança de 95%.*

O VLE foi diagnosticado em 80,24% da amostra. Todas as suas CDs, exceto “Aumento na pressão venosa central”, foram identificadas e apresentaram medidas de sensibilidade e especificidade superiores a 50%. Desse conjunto, oito CDs apresentaram alta sensibilidade (>80%); 15 apresentaram alta especificidade (>70%); e três apresentaram sensibilidade e especificidade semelhantes (>70%) (Tabela 1). As CDs com maior sensibilidade estiveram presentes em quase todas as combinações que demonstraram maior probabilidade para a presença do diagnóstico.

A prevalência de TAD foi de 54,16%. Todas as suas CDs foram identificadas na amostra. No entanto, duas delas (“Desconforto ao esforço” e “Dispneia ao esforço”) foram identificadas em todos os participantes e não compuseram a ACL final. Das cinco restantes, três apresentaram alta sensibilidade (>70%); uma apresentou alta especificidade (100%); e outra apresentou valores elevados tanto para sensibilidade quanto para especificidade (>70%) (Tabela 1). As probabilidades posteriores mostraram a importância da CD “Resposta anormal da frequência cardíaca à atividade”, pois ela compõe todas as combinações que apontam para maior probabilidade de ocorrência desse diagnóstico.

A prevalência estimada de CP foi de 44,71%. Nove CDs apresentaram alta especificidade (>90%). Por outro lado, quatro CDs apresentaram alta sensibilidade (>80%), além de três CDs apresentarem altos valores tanto de sensibilidade quanto de especificidade (>90%) (Tabela 1). Todos os conjuntos consistiram em pelo menos duas CDs de alta sensibilidade e outra de alta especificidade.

A prevalência de DCD foi de 37,22%. A baixa prevalência deveu-se à ausência das CDs desse DE na amostra. Diferentemente dos demais DEs, 11 CDs não foram identificadas na amostra, e as 25 restantes apresentaram maior especificidade. Além disso, 14 CDs também apresentaram sensibilidade maior que 50%; oito tiveram sensibilidade maior que 80%; e duas apresentaram sensibilidade maior que 90% (Tabela 1). A ACL revelou um grande número de combinações diferentes de várias CDs ocorrendo concomitantemente.

A Tabela 1 apresenta as medidas de precisão diagnósticas das CDs dos DEs estudados.

Após utilizar as probabilidades posteriores de cada DE para determinar a presença ou ausência de cada DE, a ACL da síndrome incluiu os cinco diagnósticos. A Tabela 2 apresenta as medidas de acurácia da CD “Síndrome da Função Cardíaca Deficiente” e sua prevalência. Nesse modelo, VLE e FA apresentaram os maiores valores de sensibilidade, enquanto que DCD e CP apresentaram a maior especificidade. Tanto a sensibilidade quanto a especificidade para TAD foram de aproximadamente 70%.

**Tabela 2:** Medidas de precisão diagnóstica das características definidoras da “Síndrome da Função Cardíaca Deficiente” em pessoas com insuficiência cardíaca descompensada, Brasil, 2024

Características definidoras	Se	IC95%		Esp	IC95%	
Volume de líquido excessivo	0,9072	0,8666	0,9870	0,3567	0,2487	0,5204
Débito cardíaco diminuído	0,4357	0,3631	0,5371	0,7292	0,6235	0,8344
Tolerância à atividade diminuída	0,6806	0,6115	0,7973	0,6803	0,5397	0,8527
Fadiga	0,8352	0,7756	0,8966	0,1481	0,0736	0,2235
Conforto prejudicado	0,7317	0,5803	0,9988	0,9998	0,8134	0,9999
Prevalência estimada da suposta síndrome: 61,49%						

*Se - sensibilidade; Esp - especificidade; IC95% - Intervalo de Confiança de 95%.*

A Tabela 3 mostra as probabilidades posteriores para a ocorrência da “Síndrome da Função Cardíaca Deficiente”.

**Tabela 3:** Probabilidades posteriores para a ocorrência da “Síndrome da Função Cardíaca Deficiente” em pessoas com insuficiência cardíaca descompensada de acordo com diferentes conjuntos de características definidoras, Brasil, 2024

Conjuntos	Características definidoras					n	Probabilidade posterior da síndrome	
	VLE	DCD	TAD	FA	CP		Presente	Ausente
2	Não	Não	Não	Não	Sim	1	1,00	0,00
5	Não	Não	Sim	Não	Sim	5	1,00	0,00
7	Não	Não	Sim	Sim	Sim	3	1,00	0,00
9	Não	Sim	Não	Não	Sim	1	1,00	0,00
11	Não	Sim	Não	Sim	Sim	1	1,00	0,00
13	Não	Sim	Sim	Não	Sim	1	1,00	0,00
15	Não	Sim	Sim	Sim	Sim	3	1,00	0,00
17	Sim	Não	Não	Não	Sim	5	1,00	0,00
19	Sim	Não	Não	Sim	Sim	23	1,00	0,00
21	Sim	Não	Sim	Não	Sim	7	1,00	0,00
23	Sim	Não	Sim	Sim	Sim	45	1,00	0,00
25	Sim	Sim	Não	Não	Sim	2	1,00	0,00
27	Sim	Sim	Não	Sim	Sim	20	1,00	0,00
29	Sim	Sim	Sim	Não	Sim	7	1,00	0,00
30	Sim	Sim	Sim	Sim	Não	20	0,67	0,33
31	Sim	Sim	Sim	Sim	Sim	38	1,00	0,00

*VLE - volume de líquido excessivo; DCD - débito cardíaco diminuído; TAD - tolerância à atividade diminuída; FA - fadiga; CP - conforto prejudicado; n - frequência de cada combinação dos cinco diagnósticos de enfermagem.*

Na Tabela 3, os conjuntos representam as diferentes combinações (presentes/ausentes) de cada DE (no caso da síndrome, as CDs seriam os cinco DEs investigados). Esses conjuntos foram obtidos diretamente da amostra, e sua frequência está descrita na coluna “n”. Um total de 31 combinações diferentes foram identificadas. Na tabela, foram mostradas apenas as combinações em que a probabilidade da presença da síndrome foi maior que 0,5. Além disso, as probabilidades posteriores sugerem uma forte influência do CP, pois esse DE esteve presente em quase todos os conjuntos que demonstraram maior probabilidade de ocorrência da síndrome. A maioria dos participantes identificados com a síndrome apresentou três ou mais DEs concomitantemente.

A Tabela 4 apresenta os modelos log-lineares que representam as combinações dos cinco diagnósticos que exibiram relações de dependência condicional entre eles.

**Tabela 4:** Modelos log-lineares incluindo diferentes *status* dos diagnósticos de enfermagem que compõem a “Síndrome da Função Cardíaca Deficiente” em pessoas com insuficiência cardíaca descompensada, Brasil, 2024

Parâmetro	Coef.	EP	Z	Valor de *p*	IC95%	
Constante	1,70	0,43	4,00	<0,001	0,87	2,54
[VLE = P] ^*^ [DCD = P] ^*^ [TAD = P] ^*^ [FA = P] ^*^ [CP = P]	1,95	0,46	4,27	<0,001	1,05	2,84
[VLE = P] ^*^ [DCD = P] ^*^ [TAD = P] ^*^ [FA = P] ^*^ [CP = A]	1,32	0,48	2,74	0,006	0,37	2,26
[VLE = P] ^*^ [DCD = P] ^*^ [TAD = A] ^*^ [FA = P] ^*^ [CP = P]	1,32	0,48	2,74	0,006	0,37	2,26
[VLE = P] ^*^ [DCD = A] ^*^ [TAD = P] ^*^ [FA = P] ^*^ [CP = P]	2,11	0,45	4,68	<0,001	1,23	3,00
[VLE = P] ^*^ [DCD = P] ^*^ [TAD = A] ^*^ [FA = P] ^*^ [CP = A]	1,10	0,49	2,23	0,026	0,13	2,06
[VLE = P] ^*^ [DCD = A] ^*^ [TAD = P] ^*^ [FA = P] ^*^ [CP = A]	2,04	0,45	4,51	<0,001	1,16	2,93
[VLE = P] ^*^ [DCD = A] ^*^ [TAD = A] ^*^ [FA = P] ^*^ [CP = P]	1,45	0,47	3,07	0,002	0,52	2,38
[VLE = P] ^*^ [DCD = A] ^*^ [TAD = A] ^*^ [FA = P] ^*^ [CP = A]	2,20	0,45	4,89	<0,001	1,32	3,08
[VLE = A] ^*^ [DCD = A] ^*^ [TAD = A] ^*^ [FA = P] ^*^ [CP = A]	1,27	0,48	2,62	0,009	0,32	2,21

*VLE - volume de líquido excessivo; DCD - débito cardíaco diminuído; TAD - tolerância à atividade diminuída; FA - fadiga; CP - conforto prejudicado; P - presente; A - ausente; Coef - coeficiente; EP - erro padrão; Z - teste Z; IC95% - Intervalo de Confiança de 95%.*

Conforme demonstrado na Tabela 4, nove combinações dos cinco DEs apresentaram dependência condicional. A presença concomitante dos cinco DEs indica uma forte relação entre eles.

## DISCUSSÃO

Este é o primeiro estudo a analisar se FA, VLE, TAD, CP e DCD constituem a “Síndrome da Função Cardíaca Deficiente”.

Em consonância com nossos achados, FA foi altamente prevalente na IC em outra investigação^(5)^. Isso é consistente com os mecanismos fisiopatológicos da IC de retenção de água e má perfusão tecidual, que resulta em uma percepção sustentada de cansaço e incapacidade de realizar atividades físicas e mentais habituais. No entanto, devido ao caráter subjetivo desse DE, sua identificação requer treinamento específico, além do uso de métodos de avaliação confiáveis^(13)^.

O VLE esteve presente em 80,24% dos participantes. As CDs com maior sensibilidade retratam manifestações clássicas apresentadas por pessoas com IC descompensada^(14,15)^. Essa miríade de sinais e sintomas reflete a sobrecarga de volume^(1)^. De fato, esse DE é frequentemente documentado na vigência de sintomas congestivos^(16)^, e está relacionado ao risco de piora clínica^(17)^.

A prevalência de CP foi de 44,71% em nosso estudo, o que contrasta com uma prevalência de 86% encontrada em estudo retrospectivo de 144 prontuários de idosos com IC^(18)^. Essa diferença pode ocorrer devido ao período de tempo em que os dados foram coletados dos participantes. Enquanto isso, em nosso estudo, incluímos participantes que haviam sido internados no PS por não mais de seis horas, e no estudo mencionado, os pesquisadores levaram em consideração todo o tempo de internação. O conforto físico é afetado em pessoas com IC descompensada devido ao agravamento de manifestações clínicas que impactam negativamente as atividades diárias e qualidade de vida^(6)^.

A prevalência de TAD foi de 54,16% em nossa amostra. A literatura é prolífica em demonstrar que esse DE é comum em pessoas com IC descompensada^(5,13)^. Três CDs da TAD que foram consideradas altamente sensíveis neste estudo foram críticas para fornecer evidências clínicas para apoiar esse DE em estudo anterior^(19)^. Entre essas CDs, a “Resposta anormal da frequência cardíaca à atividade” parece ter particular relevância e é atribuída ao DCD^(20)^.

O DCD apresentou a menor prevalência entre os cinco DEs. Em relação às CDs com maior sensibilidade, um inquérito brasileiro com pessoas com IC identificou cinco CDs para esse diagnóstico, corroborando os achados do presente estudo^(13)^. As CDs com maior especificidade sugerem o caráter crítico e prioritário desse diagnóstico para essa população^(14)^. No entanto, o DCD não foi identificado na maioria dos participantes desta investigação devido à variação fenotípica da IC descompensada, uma vez que alguns participantes não apresentavam sinais de má perfusão, elemento vital para a tipificação desse diagnóstico^(15)^. Da mesma forma que nossos achados, outros autores identificaram o DCD em 16% de sua amostra^(18)^. Em outro estudo recente, quatro novos fatores relacionados foram encontrados para o DCD, sustentando sua relevância na prática clínica^(21)^.

Este estudo fornece evidências de que os cinco DEs (FA, VLE, TAD, CP e DCD) ocorrem simultaneamente, sugerindo que juntos constituem a “Síndrome da Função Cardíaca Deficiente”. Esta evidência é apoiada pela forte relação entre os DEs e sua dependência condicional. De fato, a IC apresenta um comportamento multifacetado e em cascata, retratando a influência de um evento no aparecimento de outro^(22)^. Da mesma forma, uma resposta humana afetada influencia a expressão de outras respostas humanas, o que pode sinalizar a pertinência de um diagnóstico de síndrome.

A prevalência estimada de “Síndrome da Função Cardíaca Deficiente” foi de 61,49%. VLE e FA apresentaram a maior sensibilidade, que se refere à capacidade desses DEs de identificar indivíduos com a síndrome. Por serem DE altamente sensíveis, apenas alguns indivíduos com o diagnóstico de síndrome serão perdidos, e o número de resultados falsos negativos é baixo^(23)^. Por sua vez, DCD e CP tiveram a maior especificidade. Isso significa que, se esses DEs não forem identificados, é provável que o indivíduo não tenha o DE de síndrome. Como DCD e CP são altamente específicos, quando identificados, haverá poucos casos falso-positivos da síndrome.

Cumpre mencionar que a edição 2024-2026 da NANDA-I^(24)^ excluiu DCD e CP de sua estrutura, mas, ainda assim, esses DEs têm sido os mais frequentemente utilizados pelos enfermeiros^(5)^, tendo sido observadas, por meio deste estudo, expressiva prevalência de cada um deles a partir de evidências clínicas e a sua ocorrência em conjunto no contexto clínico em que foi aplicado.

### Limitações do estudo

A generalização de nossos achados está sujeita a certas limitações, e não pode ser extrapolada para todos os pacientes com IC, uma vez que o DE de síndrome foi investigado apenas em pessoas com IC descompensada e em um único centro altamente especializado. Além disso, o fato de as avaliações terem sido realizadas exclusivamente pela IP, sem validação de outros especialistas, pode ser um viés, apesar do esforço da IP em documentar com precisão os achados clínicos e a tomada de decisão. Ademais, a coleta de dados ocorreu durante a pandemia, o que pode ter influenciado os achados sobre a FA. Finalmente, foram considerados elementos da edição anterior da NANDA-I (2018-2020), publicação válida na época da realização do estudo.

### Contribuições para a área da enfermagem

Pela primeira vez na literatura, foi demonstrado que FA, VLE, TAD, CP e DCD podem constituir o DE “Síndrome da Função Cardíaca Deficiente”. Abordar as respostas humanas como uma síndrome tem várias vantagens: a síndrome pode representar com mais precisão a resposta humana à IC; gerenciar um DE de síndrome pode ser mais custo efetivo do que abordar cada DE separadamente; e um DE de síndrome facilitará a documentação de enfermagem adequada. Em relação à pesquisa, estudos futuros devem investigar a validade clínica da “Síndrome da Função Cardíaca Deficiente” em outros grupos de pessoas. Além disso, estudos para mapear os resultados sensíveis à enfermagem para esse DE e testes de intervenção devem ser realizados. Por fim, este estudo possibilitou identificar que os DEs constituintes do diagnóstico sindrômico são identificados na prática clínica, agregando evidências da necessidade de novas investigações que suportem nova inclusão dos DEs retirados da edição vigente da NANDA-I.

## CONCLUSÕES

A ocorrência simultânea de FA, VLE, TAD, CP e DCD indica uma forte relação entre eles, fornecendo fortes evidências de um DE de síndrome em pessoas com IC descompensada. Ademais, a ocorrência desses DEs em *cluster* como desdobramento de fatores causais comuns são passíveis de abordagem com intervenções de enfermagem semelhantes.

## Data Availability

Os dados da pesquisa estão disponíveis no corpo do artigo.
